# Mice Survival and Plasmatic Cytokine Secretion in a “Two Hit” Model of Sepsis Depend on Intratracheal *Pseudomonas Aeruginosa* Bacterial Load

**DOI:** 10.1371/journal.pone.0162109

**Published:** 2016-08-30

**Authors:** Damien Restagno, Fabienne Venet, Christian Paquet, Ludovic Freyburger, Bernard Allaouchiche, Guillaume Monneret, Jeanne-Marie Bonnet, Vanessa Louzier

**Affiliations:** 1 Université de Lyon, APCSe, Pulmonary and Cardiovascular Agression in Sepsis, VetAgro Sup—Campus Vétérinaire de Lyon, Marcy l'Étoile, France; 2 Hospices Civils de Lyon, Immunology Laboratory, Hôpital Edouard Herriot, Lyon, France; 3 Hospices Civils de Lyon, Service d’Anesthésie-Réanimation, Hôpital Edouard Herriot, Lyon, France; Federal University of Rio de Janeiro, BRAZIL

## Abstract

Sepsis is characterized by pro- and anti-inflammatory responses following infection. While inflammation is responsible for widespread organ damage, anti-inflammatory mediators lead to immunoparalysis increasing susceptibility to secondary infections (nosocomial pneumonia). We aimed to investigate the impact of bacterial load on survival and cytokine release in a two-hit murine (C57BL/6J) model of CLP followed by *P*. *aeruginosa* pneumonia. Plasmatic TNFα, IL-6, IL-10, sTNFr I and II were quantified until 13 days. At D5, splenocytes were processed for immunological assays or mice were intratracheally instilled with *Pseudomonas aeruginosa* (5.10^6^, 2.10^7^ and 10^8^ CFU) to evaluate survival and cytokines production. TNFα, sTNFrs, IL-6 and IL-10 increased 2h post CLP. TNFα and sTNFrs declined respectively one and two days later. In CLP mice, IL-6 and IL-10 remained high for the whole experiment, as compared to Sham. At D5, for CLP mice, whereas total T cells population (CD3+) decreased, Treg fraction (CD4+/CD25+) increased. In parallel, T cells proliferation and LPS-stimulated splenocytes ability to release TNFα decreased. At D13, survival was 100% after 5.10^6^ CFU, 50% for CLP mice after 2.10^7^ CFU and 0% for CLP and Sham after 10^8^ CFU. After instillation, IL-10 and IL-6 increased and appeared to be dose and time dependent. *Pseudomonas* was detected in all CLP and Sham’s lungs; in spleen and liver only in CLP at 2.10^7^ CFU, and in CLP and Sham at 10^8^ CFU. We demonstrated that post-CLP immunosuppression followed by *Pseudomonas aeruginosa* lung instillation increases mortality reactivates cytokines secretion and is associated with systemic dissemination in septic mice depending on bacterial load.

## Introduction

Sepsis represents a major public health concern as it remains the most common cause of death in intensive care units (ICU)[1]. Today, our knowledge about sepsis pathophysiology has grown and it is now admitted that sepsis is characterized by a dysregulation of both innate and adaptive immune responses following an infectious insult.

The first immunological response described as a result of sepsis is the massive inflammation [2] called SIRS (Systemic Inflammatory Response Syndrome). Patients with sepsis exhibit high level of circulating pro-inflammatory cytokines such as tumor necrosis factor (TNFα) or Interleukin-6 and IL-6 seems to correlate strongly with decreased survival in septic patients [3]. The failure of anti-inflammatory therapeutics to reduce patients’ mortality has questioned this paradigm. Moreover clinical observations suggest that 70% of total mortality due to septic shock occurs in a delayed fashion and are often associated with an outbreak of opportunistic pathogens [4]. It has been shown that the large population of patients surviving the initial phase of sepsis enters in an immunological state of immunosuppression and hypoinflammation called CARS (Compensatory Anti-inflammatory Response Syndrome). An elevated plasma level of anti-inflammatory cytokines such as IL-10 is another hallmark of sepsis [5]. CARS is associated not only with mortality [6], but also with the development of secondary infections [6]. If some investigators suggest a sequential evolution of SIRS to CARS, recent evidences underscore that these two responses are actually concomitant [7].

In addition to pro- and anti-inflammatory cytokines elevation, another characteristic sign of immune alteration in sepsis is T lymphocytes anergy. Lymphopenia is a well-recognized feature of sepsis [8]. Whereas total T cells population decreases, regulatory T cells fraction (Tregs, CD4^+^/CD25^+^) increases in septic patients [9]. Several functional alterations have been described such as *ex vivo* diminished ability to proliferate in response to stimulation [10].

This impaired immune response in critically ill patients makes them more likely to develop nosocomial infections. More specifically, ventilator associated pneumonia is shown in humans to be characterized by sepsis-induced immunosuppression [11]. Among these ICU-acquired infections, nosocomial pneumonias, and especially Ventilator-Associated Pneumonias (VAP) are a leading cause of mortality among critically ill patients such as in sepsis [12]. *Pseudomonas aeruginosa* is the most frequently encountered multi-drug resistant Gram-negative bacterium causing VAP and nosocomial pneumonias [13]. Patients suffering from *P*. *aeruginosa* pneumonia are also more likely to develop multiple organ failure and to die than patients with other type of pneumonias [14]. One explanation for the poor prognosis associated with *P*. *aeruginosa* pneumonia is that some strains cause acute lung injury and disseminate into the circulation [13,15].

Therefore sepsis appears as a “two-hit” aggression where two different temporal patterns (*ie* early and late) can be depicted. The initial event primes the host in such a way that a later insult can lead to a synergistic response disproportionate to the severity of the insult [16]. So, two injuries relatively innocuous separately may have dramatic repercussions if associated together in a sequential way over a short period. To explore sepsis pathophysiology, there are multiple experimental two-hit models that mimic what might be seen in clinics. Although most animal models are not directly relevant to investigate human sepsis pathophysiology, appropriate animal models remain crucial either to understand the course of sepsis or to develop new therapies. Among all mice models, the Cecal Ligation and Puncture (CLP), a mouse model of peritonitis, closely replicates the clinical picture encountered in human patients and has become, as a gold standard model, the most frequently used model of sepsis [17–19]. Nevertheless, the way to perform CLP, the possible adjuvant therapeutics as in human sepsis (antibiotics, resuscitation, analgesia), the gender or the strain of the mouse can influence and change the characteristics of the model.

The purpose of this study was to investigate how different intratracheal bacterial load of *P*. *aeruginosa* can influence surviving and cytokine release in previously septic mice. This two-hit model of CLP followed by *P*. *aeruginosa* pneumonia mimics a situation frequently encountered in septic patients. Evaluating the consequences of different doses of *P*.*aeruginosa* on lethality and cytokines production would allow us to understand potential mechanisms through which this combination of insults induces mortality.

## Material and Method

### Double hit model of sepsis: Sepsis followed by pneumonia

#### Mice

**Fig 1:**
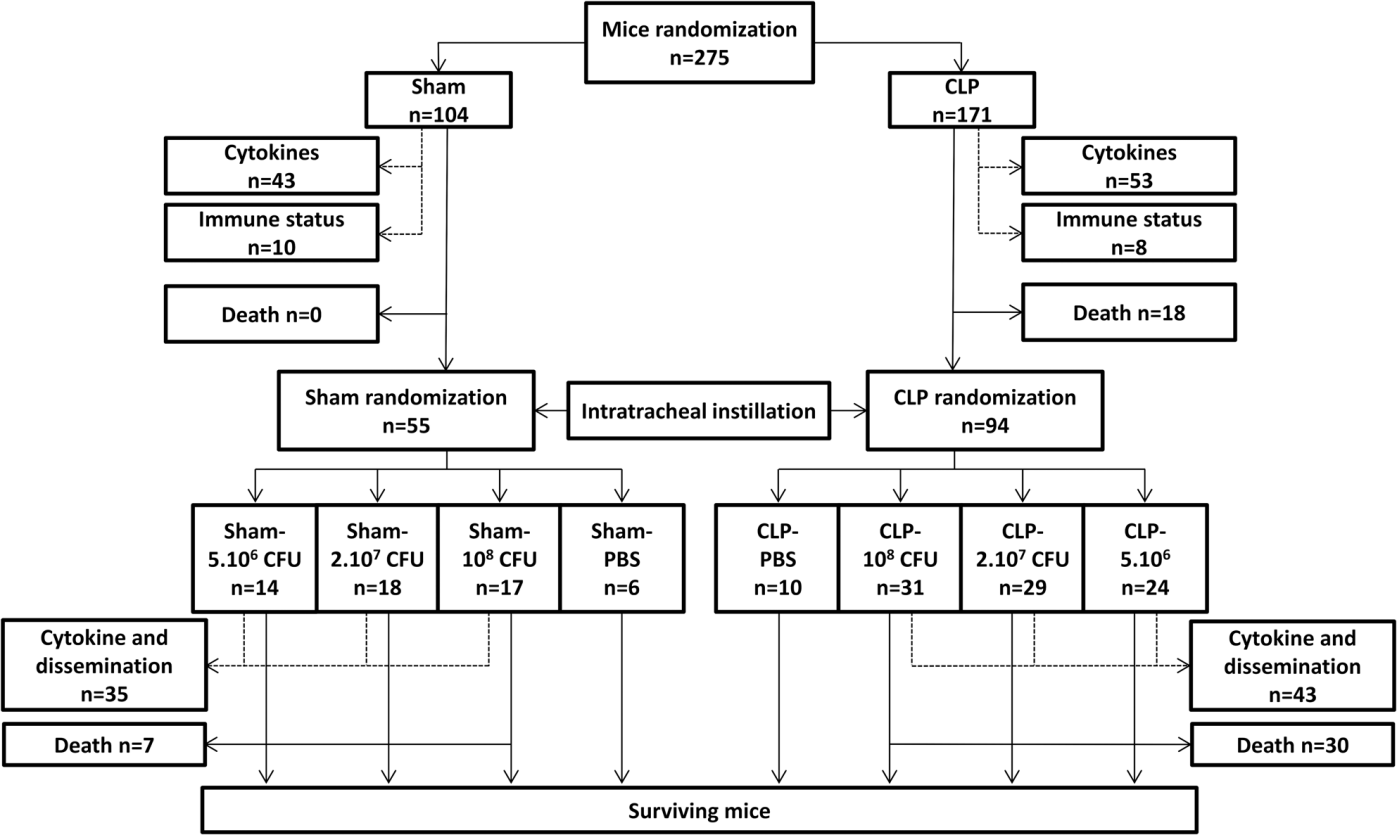
Study design flowchart. Mice were randomized into 2 groups: Sham or CLP. In each group, some mice were used to evaluate cytokines levels and immune status (dotted arrows) and were therefore not included in the survival evaluation after the first septic insult. Note that some mice were used for both cytokine assessment and immune status evaluation. The second stage started right after the intratracheal administration of *Pseudomonas aeruginosa*. Sham and CLP groups mice were randomized into 4 different groups according to the amount of bacteria they received: Sham- or CLP-PBS were instilled with PBS (vehicle) only; other groups were instilled with the different bacterial loads as follow: Sham- or CLP-5.10^6^ CFU, Sham- or CLP-2.10^7^ CFU and Sham- or CLP-10^8^ CFU. Systemic cytokine release and spleen and liver bacteria dissemination were evaluated in the same mice. These mice were not included in the survival evaluation of the second hit. “n” represent the animal number in each condition.

Male C57BL/6J mice (Charles River, L’Arbresle, France), 7 to 9 weeks of age (20-25g) were housed during one week before the experiments in a conventional GLP facility, with a 12 hours dark/light cycle. Mice (total n = 275) were divided into several groups (see flowchart on Fig 1 for groups and animal numbers). Briefly, the first insult involved two groups after mice randomization: Sham animals and CLP animals. Then after the intratracheal instillation each group was divided once again into 4 different groups after new randomization.

#### Ethic statement

All the experiments were approved by the Institutional Animal Care and Use Committee at VetAgro Sup, Lyon, France (proposal 1403) in accordance with European Convention for the Protection of Vertebrate Animals used for Experimental and other Scientific Purposes.

For survival studies the time of death is the most critical endpoint. In studies as sepsis euthanizing mice prematurely might false the results. Nevertheless, minimizing animal distress or suffering is of prime importance. Therefore, a balance between minimizing animal pain and avoiding bias in survival curves should be found. Animals’ wellbeing was followed every 8 hours for signs of distress and endpoints. The overall health status was checked by trained professionals (*e*.*g*. DVMs). Specific criteria used to determine when the animals should be euthanized were based on personal experience and in accordance with Remick lab report [20]. Mice were systematically euthanized when they were found in a moribund state as identified by inability to maintain upright associated or not with labored breathing and cyanosis. Classical signs of distress such as anorexia and weight loss (> 20%), hunching, prostration, impaired motility, labored breathing, ruffled haircoat, dehydration, were assessed. Mice exhibiting at least four of these criteria were humanely euthanized via isoflurane (5%) anesthesia followed by cervical dislocation. Mice exhibiting less than four of these criteria were evaluated again 8 hours later: then if mice worsened they would be euthanized. Euthanized mice were considered as non-survivors. These endpoints criteria were used for both CLP and secondary infections. In parallel analgesia was performed with buprenorphine, an opioid medication, because CLP is classified as a severe procedure. Buprenorphine (Buprecare, Axiences SAS, Pantin, France: 0.5mg/kg) was subcutaneously injected before surgery and after the insult, twice daily for the next two days.

#### First hit: Model of polymicrobial sepsis (CLP)

A sublethal polymicrobial sepsis was induced by Cecal Ligation and Puncture as previously described [21]. Right after a subcutaneous injection of buprenorphine (Buprecare, Axiences SAS, Pantin, France: 0.5mg/kg), mice were anaesthetized with isoflurane inhalation (induction 5% and maintenance 2%) and the abdomen was shaved and disinfected. The cecum was identified and exteriorized thanks to a midline laparotomy, ligatured at its external third (30% ≈ 0.8 to 1cm depending on the caecum size), and punctured twice with a 21-gauge needle to create two single holes (this is not a through and through puncture). After removing the needle, a small amount (droplet) of feces from penetration holes was extrude to ensure patency. The cecum was replaced in the abdominal cavity. Incision was sutured in layers and animals were resuscitated with an intraperitoneal (I.P.) injection of 0.6 ml of saline (See S1 Fig for the conduction of CLP). Controls were Sham-operated mice undergoing laparotomy with only exposition of cecum without CLP. Six hours following surgery and then every 12 h for the next two days, mice received an I.P. injection of antibiotics (Imipenem cilastatine, Tienam, Merck Sharp and Dohme, 25 mg/kg in 0.6 ml of saline). Pain was controlled by subcutaneous injection of buprenorphine (0.5mg/kg) given 5–6 hours post CLP and twice daily for the next two days.

### Second hit: Induction of secondary *Pseudomonas aeruginosa* pneumonia

#### Bacteria preparation

*Pseudomonas aeruginosa* (ATCC® 27853™) was grown in a 9 ml BHI broth (BioMerieux, Marcy l’Etoile,France) for 24 hours. Then 200μl of this bacterial suspension were grown again in 9 ml BHI for 15 additional hours at 37°C to reach bacterial exponential growth. Bacteria were washed twice and diluted in PBS, and the desired concentration was adjusted by spectrophotometry (absorbance/OD at 600 nm): CFU were extrapolated according to a reference curve (0.045 _A600_, 0.234_A600_ and 0.61_A600_ corresponding to 5.10^6^, 2.10^7^ and 10^8^ CFU/50 μl respectively). After instillation, real CFU measurement was systematically verified by quantitative culture (24h, 37°C) of the bacterial inoculum.

### Intratracheal instillation and survival

**Fig 2:**
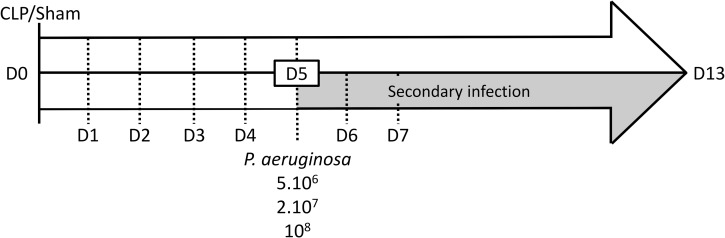
Study protocol. Mice were Sham- or CLP-operated at D0. Five days after surgery (D5), mice were or were not intratracheally instilled with *P*. *aeruginosa*. Plasmatic cytokines were measured at 2h, 6h, D1, D2, D3, D5 and D13 and also at D6 and D7 only for instilled mice.

To ensure mortality is not CLP-dependent anymore, five days post-CLP (D5), surviving mice received 50 μl of PBS intratracheally (Sham n = 6, CLP n = 10). To assess the effects of bacterial load on mortality the other surviving mice were randomized in 3 groups receiving 50 μl of *Pseudomonas aeruginosa* at different doses: 5.10^6^ (Sham n = 6, CLP n = 15), 2.10^7^ (Sham n = 10, CLP n = 17) and 10^8^ CFU (Sham n = 7, CLP n = 20). For intratracheal instillation, short-duration anaesthesia was induced by isoflurane inhalation. Briefly, mice were placed in the supine position on a 60° incline board by holding their upper incisor teeth. Their tongue was gently pulled outside to allow the access to the pharynx. 50 μL of the bacterial suspension was instilled at the back of the oral cavity above the tracheal opening and mice nose was blocked to induce a forced inspiration so the suspension was literally sucked into the lungs. Survival was evaluated over 8 days (Fig 2).

### Immune status

#### Plasmatic cytokines quantification after CLP

Cytokines were measured in CLP mice without secondary infection. Mice were sacrificed at different times and blood was collected 2h (Sham n = 7, CLP n = 8), 6h (Sham n = 7, CLP n = 9), one (D1: Sham n = 7, CLP n = 10), two (D2: Sham n = 7, CLP n = 10), three (D3: Sham n = 7, CLP n = 7), five (D5: Sham n = 4, CLP n = 4) and thirteen days (D13: Sham n = 4, CLP n = 5) after surgery in K3 EDTA tubes *via* the submandibular vein to quantify circulating cytokines (Fig 2). After secondary infection mice were sacrificed one (D6 Sham/CLP: 5.10^6^ CFU n = 4/4; 2.10^7^ CFU n = 4/5; 10^8^ CFU n = 5/5), two (D7 Sham/CLP: 5.10^6^ CFU n = 4/5; 2.10^7^ CFU n = 4/5; 10^8^ CFU n = 5/5) and eight days (D13 Sham/CLP: 5.10^6^ CFU n = 4/5; 2.10^7^ CFU n = 5/6) after intratracheal instillation (Fig 2).

Plasmatic pro-inflammatory TNFα and IL-6, along with anti-inflammatory IL-10 were quantified with the multiplex MCYTOMAG-70K assay (Millipore, Molsheim, France). Soluble TNF Receptor I and II (sTNFr-I and sTNFr-II respectively) were assessed using the multiplex MSCRMAG-42K assay (Millipore, Molsheim, France) according to manufacturer’s instructions.

#### Splenocytes culture

Prior to secondary infection, immune status was investigated on 18 mice (10 Sham and 8 CLP): on the 5^th^ day (D5), which is the day of the secondary challenge, mice splenocytes were collected, isolated and cultured in RPMI 1640 (Eurobio, Les Ulis, France).

#### Lymphocytes phenotyping

Total T cells population was determined from cultured and isolated splenocytes using flow cytometry (FC500 Beckman Coulter). Isolated cells were marked with an anti-CD3+ antibody (PC5 Hamster Anti Mouse CD3e (145-2C11), BD Biosciences, Mountain View, CA) to quantify total lymphocytes population. T regulatory cells (Treg) were also quantified thanks to an anti-CD4+/anti-CD25+ antibody (PC7 Rat Anti Mouse CD4 (Clone RM4-5)—PE Rat Anti Mouse CD25 (Clone PC61), BD Biosciences, Mountain View, CA). Absolute counting was performed using calibrated beads (Flow counts beads, Beckman coulter).

#### Lymphocytes proliferation assay

Splenocytes were cultured (200 000 cells/well) for three days with an anti-CD3 antibody at 1μg/ml (Purified NA/LE Hamster Anti Mouse CD3e (145-2C11), BD Biosciences, Mountain View, CA). Then, [^3^H] thymidine was added in the culture 24 h before harvesting cells on a fiberglass filter using an automated cell harvester (PerkinElmer). Incorporated radioactivity was measured in a direct beta counter (PerkinElmer,count per minute). Results are expressed as proliferation ratios between counts per minutes measured in stimulated and non-stimulated wells for the same experiment.

#### Splenocytes TNFα expression

Splenocytes were cultured and stimulated with LPS for 24h (200 000 cells/well, LPS 1μg/ml [22]). TNFα production was quantified in the culture supernatant using ELISA according to the manufacturer’s instruction (BD Biosciences, Mountain View, CA).

### Assessment of lung infection and systemic dissemination:

Five days after CLP or Sham surgery mice were intratracheally instilled with three different doses of *P*. *aeruginosa* (5.10^6^, 2.10^7^ and 10^8^ CFU).Pneumonia was confirmed histologically (See S2 Fig). Bacteremic dissemination was indirectly assessed through quantitative spleen or liver bacterial cultures on selective medium. Two days (D7 Sham/CLP: 5.10^6^ CFU n = 6/7; 2.10^7^ CFU n = 9/12; 10^8^ CFU n = 5/11) or eight days (D13 Sham/CLP: 5.10^6^ CFU n = 4/5; 2.10^7^ CFU n = 5/6) after induction of pneumonia, lungs, spleen and liver were removed and mechanically homogenized in PBS under sterile conditions. Organ homogenates were subjected to serial 10-fold dilutions and cultured for 24 hours on Tripcase Soja and *Pseudomonas* Agar Base + CFC (cetrimide, fucidin, cephalosporin) plates (BioMerieux, Marcy l’Etoile,France). API 20E (Analytical Profile Index System for Identification of Enterobacteriaceae and gram negative bacteria, BioMerieux, Marcy l’Etoile,France) was used for the identification of the bacteria.

### Statistical analysis

Cytokines results were analyzed and compared longitudinally using linear models. All cytokine measures were log-transformed to better meet the normal distribution. Models were selected according to the lowest Akaike Information Criterion (AIC). After surgery, cytokines comparisons were performed using ANOVA on a linear model with interaction. Sham vs CLP comparisons were performed varying time levels among the linear interaction model and corrected p value were given according to the Bonferroni method. Cytokines quantities from the second phase (after secondary pulmonary infection) were analyzed with a generalized linear model to test the relationship between dose, time and group.

Other results are reported as means ± SEM. Immunological changes and organ bacterial counts were compared by non-parametric Mann-Whitney or Kruskal-Wallis tests. Survival curves (Kaplan–Meier plots) were compared by log rank test. P values < 0.05 were considered statistically significant.

Analyses were performed using the software R 2.15.2.

## Results

### Plasmatic cytokines after CLP

7 days after acclimatization and 7 days before surgery, TNFα, IL-6 and IL-10 were undetectable whereas sTNFr-I and sTNFr-II were 3.31 ± 0.05 log(pg/ml) and 3.733 ± 0.021 log(pg/ml), respectively.

After surgery, data analysis showed: i) a group effect (p<0.0001) as CLP operated animals exhibited higher amounts of cytokines; ii) a time effect (p<0.0001) as plasmatic cytokine levels decreased over 13 days; iii) an interaction between groups and time (p<0.01).

**Fig 3:**
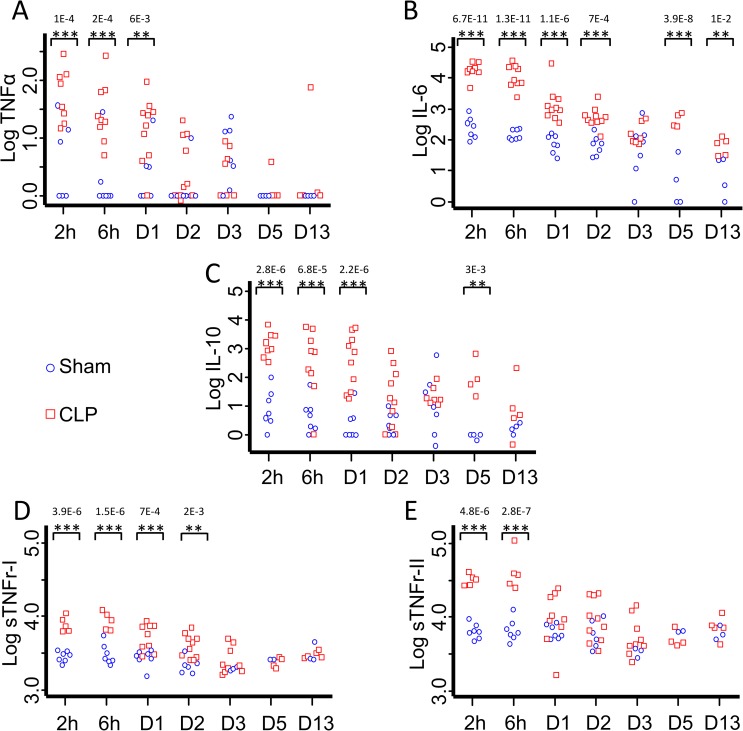
Plasmatic cytokines assessment after CLP. Plasma was collected 2 and 6 hours, 1 (D1), 2 (D2), 3 (D3), 5 (D5) and 13 (D13) days after CLP or Sham surgery with no secondary infection to measure TNFα, IL-6, sTNFr I and II and IL-10 concentrations thanks to the Luminex technique. At D0, before surgery, TNFα, IL-6 and IL-10 were undetectable. Both sTNFr were detectable in healthy mice. Pro-inflammatory cytokines TNFα (A) and IL-6 (B) are significantly increased as soon as 2h after sepsis induction, as anti-inflammatory mediators IL-10 (C) and sTNFr-I and II (D and E). TNFα was not detected anymore in Sham operated mice at D5 and D13. Results are expressed as Log(pg/ml). p values given are corrected p values according to the Bonferroni correction. *CLP *vs* Sham p<0.05, **CLP *vs* Sham p<0.01, ***CLP *vs* Sham p<0.001.

In Sham operated mice, IL-6 and IL-10 became detectable from 2h to 13 days while TNFα remained detectable only until D3 (Fig 3).

The pro-inflammatory cytokine TNFα was significantly increased in CLP mice as compared to Sham at 2 hours (11 fold higher, 89.364±32.47 *vs* 8.469±5.174 pg/ml; p<0.001), 6 hours (13.5 fold, 55.33±28.19 *vs* 4.31±4.03 pg/ml; p<0.001) and 24 hours (8 fold, 24.89±8.66 *vs* 3.9±2.822 pg/ml; p<0.01) post-surgery. Then, TNFα amounts decreased but remained detectable until D13 in CLP-operated mice (Fig 3A).

IL-6 levels significantly increased as soon as 2 hours after surgery and remained significantly higher in CLP as compared to Sham from 2 hours (60 fold higher, 20028.52±3356.829 *vs* 329.32±100.04 pg/ml p<0.001) to 13 days (5 fold, 71.97±18.62 *vs* 16.21±6.36 pg/ml; p<0.01) (Fig 3B).

Anti-inflammatory cytokine IL-10 was massively released after the CLP procedure and remained significantly higher than in Sham operated mice until D5 (100 fold higher at 2 hours (2081.97±756.452 *vs* 21.2±12.81 pg/ml; p<0.001) and 30 fold at Day 13 (44.51±40.26 vs 1.48±0.537 pg/ml: p<0.001)) (Fig 3C).

sTNFr-I and sTNFr-II had a similar evolution during our experimental sepsis. Their concentrations were significantly higher after surgery as compared to seven days before surgery (sTNFr-I: D-7: 2.06 ± 0.23 ng/ml and 2hours post CLP: Sham 2.90 ± 0.18 ng/ml, CLP 8.01 ± 0.91 ng/ml / sTNFr-II: D-7: 5.41 ± 0.26 ng/ml and 2hours post CLP: Sham 6.44 ± 0.60 ng/ml, CLP 31.60 ± 2.54 ng/ml). sTNFr-I and sTNFr-II concentrations were also significantly higher in CLP as compared to Sham operated animals, at 2hours (2.8 / 4.9 fold higher respectively; p<0.001 for both), 6 hours (2.8 / 6.8 fold higher respectively; p<0.001 for both) and, for sTNFr-I only, at 1 and 2 days after CLP (2 and 1.9 fold higher respectively; p<0.001) (Fig 3D and 3E).

### T cells population decreases while Treg fraction increased

**Fig 4:**
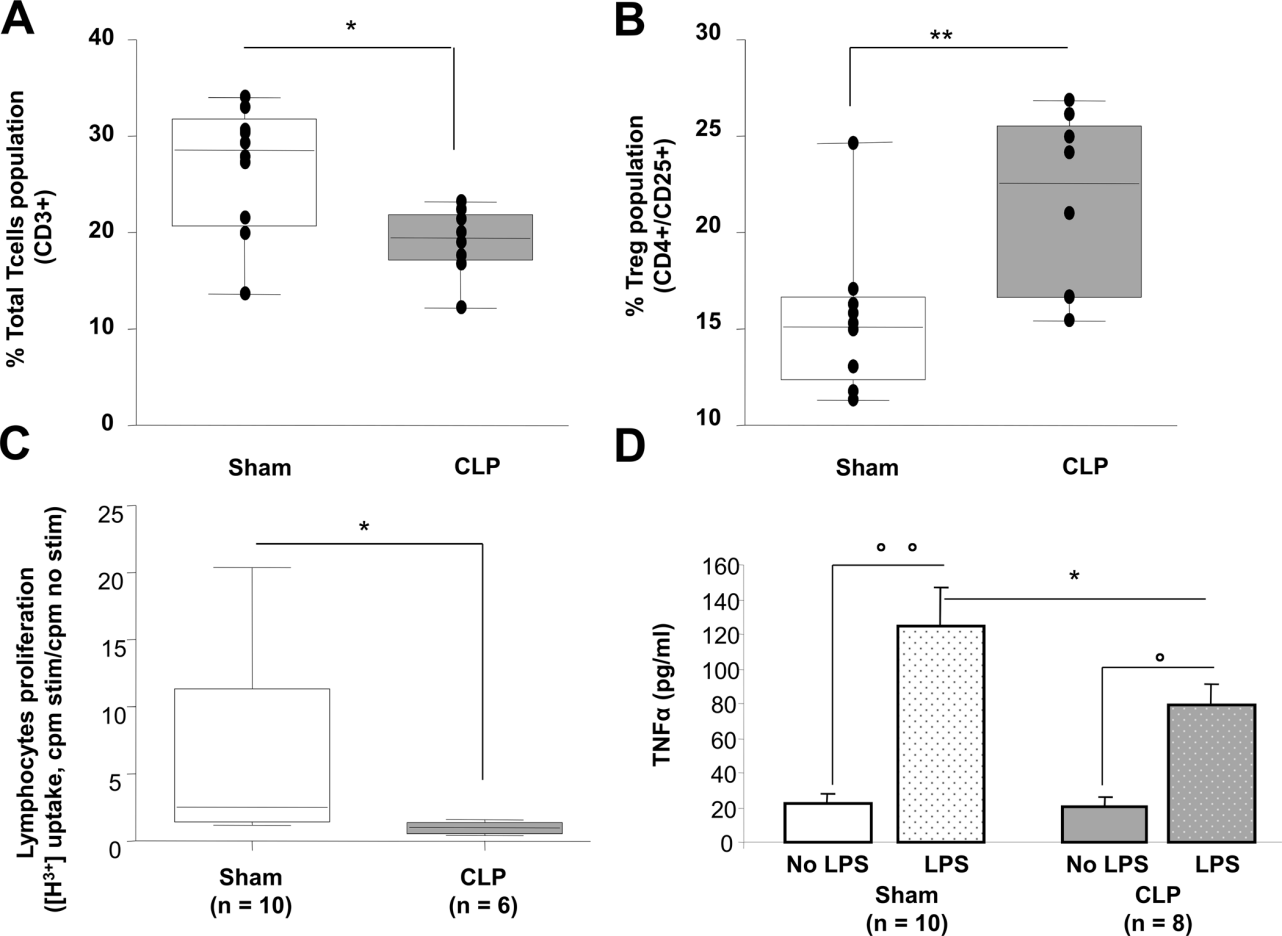
Immunological assay. Spleens were harvested at D5 and splenocytes were rapidly isolated in order to determine T cells characteristics (population, proliferation, and TNFα secretion). As compared to Sham-operated animals (n = 10), total T cells population (CD3+) in CLP mice (n = 8) decreased (A) among which Treg fraction (CD4^+^/CD25^+^) increased (B). In parallel, T cells proliferation, assessed by a stimulation with an anti-CD3^+^ antibody (200 000 splenocytes / anti CD3 coated wells (1**μ**g/ml)) and incorporation of [^3^H]-thymidine was diminished (C). Radioactivity counts reflect the lymphocytes proliferation. Results are expressed as proliferation ratios between counts per minute measured in stimulated (cpm stim) versus non-stimulated (cpm no stim) wells for the same experiment. Splenocytes ability to produce TNFα when stimulated for 24 hours with LPS (200 000 cells/well, LPS 1**μ**g/ml) was also decreased (D). *p<0.05 Mann Whitney U test, **p<0.01 Mann Whitney U test, °p<0.05 Wilcoxon paired test °°p<0.01, Wilcoxon paired test.

Total splenic T cells population (T-CD3+) decreased in CLP mice as compared to Sham 5 days after operation (19.0 ± 1.2% *vs* 26.7 ± 2.0% respectively, p<0.05, Fig 4A) whereas T regulatory cells fraction (Treg, T-CD4+/CD25+) significantly increased (21.46 ± 1.65% *vs* 15.3 ± 1.2% respectively, p<0.01, Fig 4B). In addition with a reduced percentage, the absolute count of T cells was reduced as well after CLP: 6608 ± 1464 T cells/μl were counted in spleens from sham mice versus 4380 ± 1084 T cells/μl in septic mice. This was also observed for CD4+ T cells (3171 ± 766 CD4+ T cells/μl in Sham mice versus 1860 ± 492 CD4+ T cells/μl in septic mice). On the contrary, the absolute count of Treg was not modified (447 ± 105 versus 377 ± 78 cells/μl).

### Lymphocytes proliferation

5 days after the septic assault, splenic lymphocytes proliferation was dramatically impaired (4.4 fold decrease) as [^3^H] thymidine uptake was significantly lower (p<0.05) in CLP T cells as compared to Sham T cells (Fig 4C).

### Polymicrobial sepsis alters splenocytes ability to release TNFα

Sepsis-induced production and release of pro-inflammatory TNFα by isolated splenocytes, cultured and stimulated for 24h with LPS, was measured five days post CLP. Splenocytes coming from CLP-operated mice released lower amounts of TNFα as compared to Sham’s splenocytes after LPS stimulation (79.53 ± 11.63 pg/ml *vs* 125.05 ± 21.76 pg/ml respectively, p<0.05, Fig 4D).

### Effect of CLP and *Pseudomonas aeruginosa* intratracheal instillation on survival

**Fig 5:**
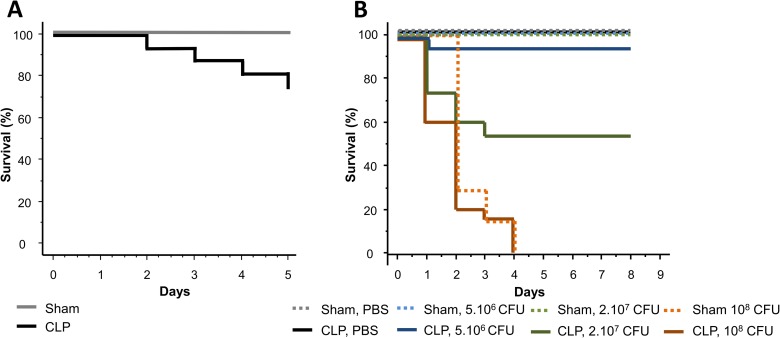
Survival after bacterial challenges. (A) During the first septic assault, after CLP and prior to *P*. *aeruginosa* instillation (D5), 28% of CLP-operated mice (22/80) died. During this period, 100% of Sham-operated mice (n = 29) survived. (B) At D5 post CLP, mice were challenged with an intratracheal administration of *P*. *aeruginosa*. Since CLP mice died until 5 days, final survival data were given by subtracting lethality induced by CLP during the first assault. So we considered the remaining living mice as 100% for this second survival study. Either PBS (Sham n = 6, CLP n = 10) or *Pseudomonas aeruginosa* were instilled. For bacterial challenge, three doses were administered: 5.10^6^ CFU (Sham n = 6 and CLP n = 15), 2.10^7^ CFU (Sham n = 10 and CLP n = 17) and 10^8^ CFU (Sham n = 7 and CLP n = 20). Results are expressed as Kaplan Meier survival curves. p < 0.05 were considered statistically significant.

In the first five days following CLP and before the intratracheal bacterial challenge (at D5), 28% of CLP mice died (Fig 5A) while every Sham-operated mice survived. After 5 days, there was no more mortality of CLP mice (Fig 5B black and grey color lines): final survival data were given by subtracting lethality induced by CLP during the first assault.

After intratracheal instillation of *Pseudomonas aeruginosa* (at D5) survival depended on bacterial load. With the lowest dose of bacteria (5.10^6^ CFU), only one CLP mouse died (survival = 93%) while all Sham mice survived. Increasing the dose led to an enhanced mortality (p<0.001). At 2.10^7^ CFU only 53% of CLP mice died whereas all Sham operated mice survived (p<0,01). At an even stronger dose, 10^8^ CFU, both Sham and CLP mice died rapidly. Then, 4 days post-intratracheal instillation there were no more mice alive (Fig 5B).

### Plasmatic cytokines after *P*. *aeruginosa* pulmonary challenge

**Fig 6:**
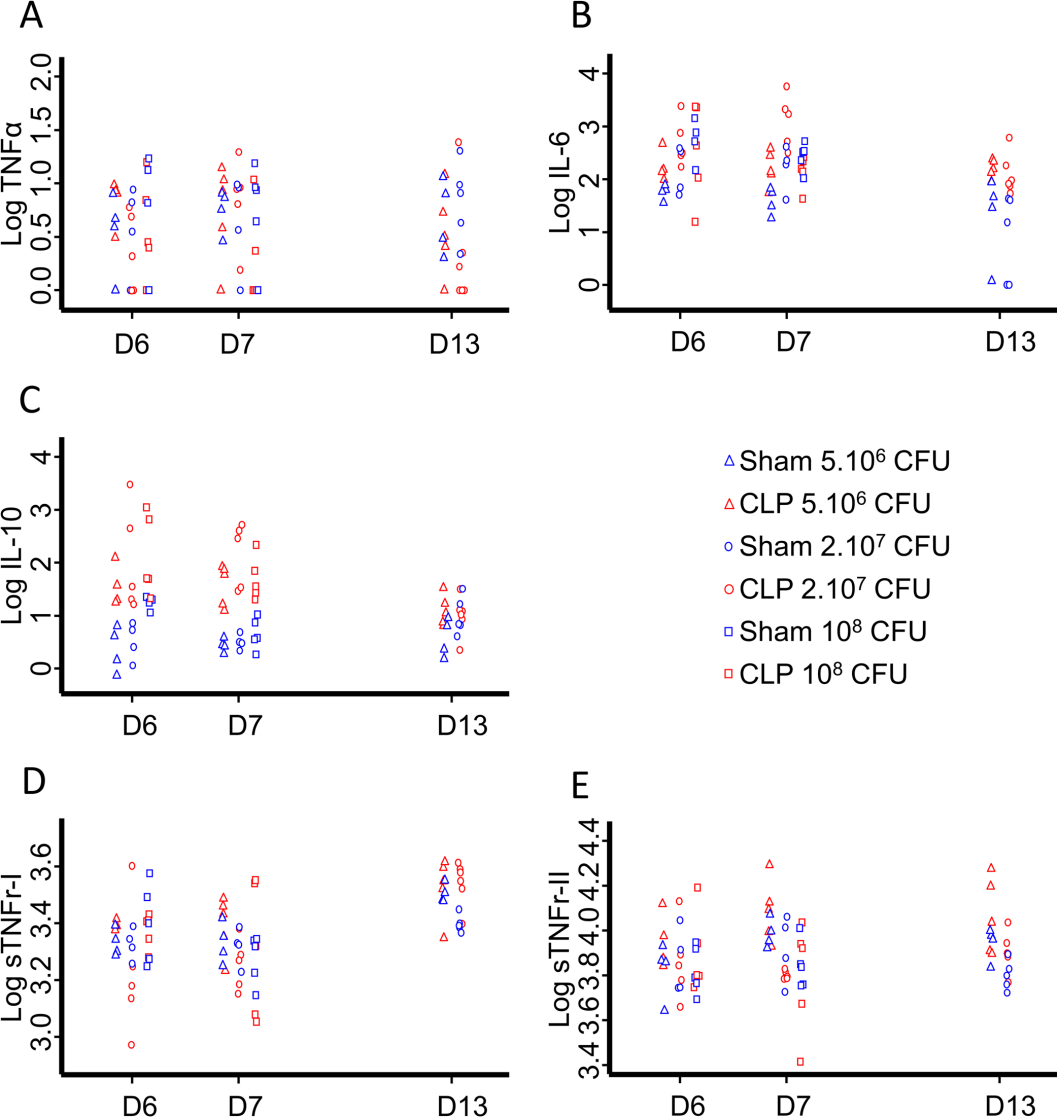
Plasmatic cytokines assessment after bacterial challenges. Plasma was collected 1 (D6), 2 (D7) and 8 (D13) days after *P*. *aeruginosa* intratracheal instillation and TNFα, IL-6, sTNFr I and II and IL-10 were measured thanks to the Luminex technique (n≥4 for each groupe or dose). While no group, nor time or dose effect could be evidenced for TNFα (A), IL-6 and IL-10 levels were dose-dependent and decreased over time (B and C). No group effect but a dose-dependent decrease of sTNFr-I (D) at D13 and sTNFr-II (E) at D6, D7 and D13 were observed. sTNFr-I increased over time whereas sTNFr-II did not. Results are expressed as Log(pg/ml). Effects are considered significant when p<0.05.

TNFα, IL-6, IL-10 and sTNFr were detectable in both Sham and CLP mice either one (D6), two (D7) or eight (D13) days after *P*. *aeruginosa* intratracheal instillation (Fig 6).

After this secondary challenge, no group effect, nor time effect or dose effect could be evidenced for TNFα (Fig 6A).

Plasmatic IL-6 and IL-10 concentrations were time-dependent and decreased over 8 days (p<0.001 for both cytokines). CLP operated animals exhibited higher IL-6 (p<0.001) and IL-10 (p<0.001) amounts as compared to Sham. A bacteria dose-dependent effect was observed: IL-6 and IL-10 were higher for 10^8^ CFU than for 5.10^6^ CFU (p<0.01 and p<0.05 respectively), and for 2.10^7^ as compared to 5.10^6^ (p<0.05 and p<0.05 respectively). An interaction between groups and time was found only for IL-10 eight days after *P*. *aeruginosa* infection (D13; p<0.01, Fig 6B and 6C).

No group effect was observed for plasmatic sTNFr-I and sTNFr-II. sTNFr-I increased over 8 days (p<0.001) whereas sTNFr-II remained stable. A bacteria dose-dependent effect was observed: sTNFr-I at D13 (p<0.05) and sTNFr-II at D6 (p<0.05), D7 (p<0.05) and D13 (p<0.001) were higher for 5.10^6^ CFU than for 2.10^7^ CFU (Fig 6D and 6E).

### Bacterial dissemination

**Fig 7:**
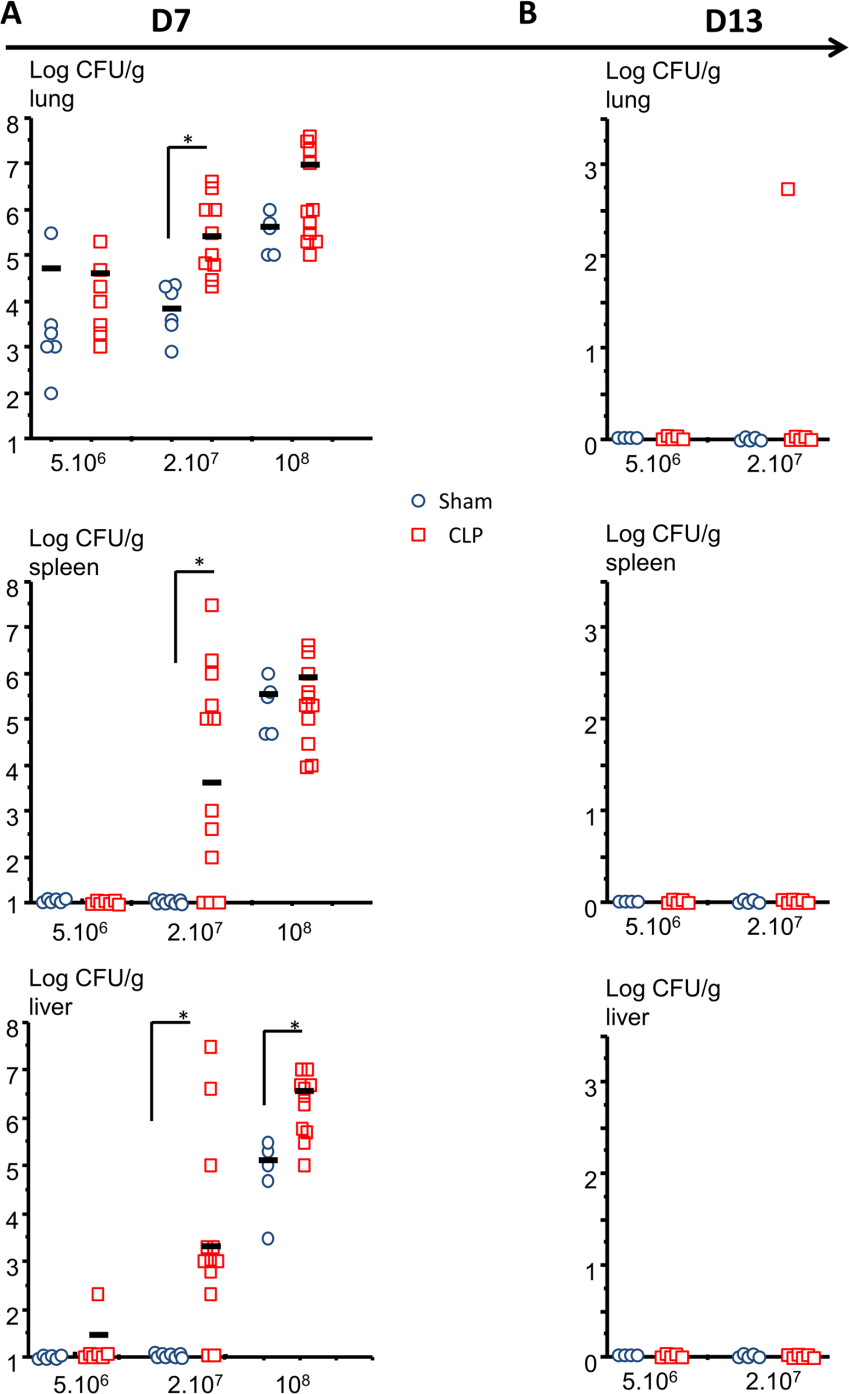
Bacterial dissemination. 5 days after surgery (D5), mice were intratracheally instilled with 5.10^6^ CFU, 2.10^7^ CFU or 10^8^ CFU of *Pseudomonas aeruginosa* and their lungs, liver, spleen were harvested 2 (D7; A) or 7(D13; B) days later, grinded, diluted and cultured for the presence of *P*. *aeruginosa*. The results are expressed as log CFU/g tissue. *p<0.05 Mann Whitney U Test.

Two days after intratracheal instillation (D7), *Pseudomonas aeruginosa* was present in all CLP and Sham mice lungs. *Pseudomonas* CFU were significantly higher in CLP (9.6.10^5^ ± 1.4.10^5^ CFU) than in Sham operated mice (1.2.10^4^ ± 0.17.10^4^ CFU) (Fig 7A).

*Pseudomonas* was not detected in Sham operated mice liver or spleen at 5.10^6^ CFU and 2.10^7^ CFU but at 10^8^ CFU. In CLP operated mice, 1/7 mice at 5.10^6^ CFU and 9/12 mice at 2.10^7^ CFU exhibited *Pseudomonas* in their liver and 0/7 mice at 5.10^6^ CFU and 8/12 mice at 2.10^7^ CFU in their spleen. At 10^8^ CFU all mice exhibited bacteria either in their liver or in their spleen but CLP mice had significantly greater amounts of *Pseudomonas* in their liver as compared to Sham (Fig 7A).

7 days after intratracheal instillation, only 1/6 CLP mice initially injected with 2.10^7^ CFU continued exhibiting *Pseudomonas* in its lungs. Neither the livers nor the spleens were positive for this germ in both Sham and CLP mice (Fig 7B).

## Discussion

Sepsis is characterized by a complex pathophysiology. Thus, understanding its different aspects and its evolution throughout time represents a major issue for physicians and scientists to improve the care and management of ICU patients. In this study, we aimed to understand the impact of *Pseudomonas aeruginosa* bacterial load on mortality after pulmonary secondary infection during sepsis. To do so, we characterized the pathophysiology of our model and linked it to the human sepsis clinical picture. Although most animal models are not directly relevant to investigate human sepsis, they remain essential to understand the pathophysiology of sepsis or to develop new therapies. CLP is, to date, the best model that can represent the complexity of human sepsis. CLP combines several advantages: the procedure is straightforward and reproducible in the lab, supportive therapies are possible, procedure does not induce only local peritonitis but also bacterial dissemination, and plasmatic cytokine response is similar to that observed in septic patients. Inconvenient of such a procedure are the mismatch between human and animal regarding age and sex, the high n-numbers required, the variability due to the strain and the necessity to really standardize the procedure to compare studies [23]. CLP-induced mortality depends on several technical parameters such as the position of the ligation, the needle size and the number of cecal punctures. Fluid resuscitation and broad spectrum antibiotherapy also influence the outcome of CLP [23]. We chose to standardize our model as follow. We performed a mild cecal ligation and puncture (30%, 2 punctures with a 21G needle) in order to have low mortality and to induce the first septic assault of our two-hit model of sepsis. To mimic human sepsis management, mice are resuscitated with fluids, treated with antibiotics and analgesia is performed. The analgesic management is made with buprenorphine because it has no effect on mortality as long as the dose is low and has minimal effects on immune parameters [24]. It has been repeatedly demonstrated that antibiotics improve survival in both human studies and animal research models of sepsis [25,26]. Imipenem, the antibiotic used in our model, is a broad-spectrum carbapenem antibiotic combination so that the secondary infection occurs in a ground exempt of *Pseudomonas aeruginosa*. Following the first septic assault, to simulate secondary mechanical ventilation-induced pneumonia, we chose to intratracheally instill live *Pseudomonas aeruginosa*. Indeed, most patients with sepsis do not die of primary infection since over 70% of total mortality after septic shock occurs in a delayed fashion. Thus, mortality is rather a result of the combination of abolished immune reactions and secondary infection, most commonly ventilator-associated pneumonia, and *Pseudomonas* is the most frequently incriminated bacteria [12].

Our model of sepsis displays early and massive inflammation as TNFα and IL-6 rise significantly (60-fold higher concentration as compared to Sham for both these cytokines at the second hour post CLP). These two cytokines increase during the earlier stages after CLP (from 2 hours) and remain higher in septic mice as compared to Sham until 24 hours for TNFα and 13 days for IL-6. Our model exhibits the same early increase of anti-inflammatory molecules such as soluble TNF receptors (sTNFr: almost 9-fold increase) and IL-10 (100-fold increased). sTNFr-II and sTNFr-I remain higher in septic mice as compared to Sham until 6 or 24 hours respectively whereas IL10 decreases slowly over time. Interestingly sTNFr evolve in parallel with TNFα plasmatic concentration. In our study, both pro- and anti-inflammatory mediators increase in the early stage after the septic injury. This increase is transient for TNFα and sTNF receptors but persists for several days for IL-6 and IL-10. Regarding TNFα, IL-6, IL-10, sTNFr, our results confirm that both pro-inflammatory and anti- inflammatory responses occur early and simultaneously in human sepsis [27] as well as after mild CLP procedure in mice [7].

**Fig 8:**
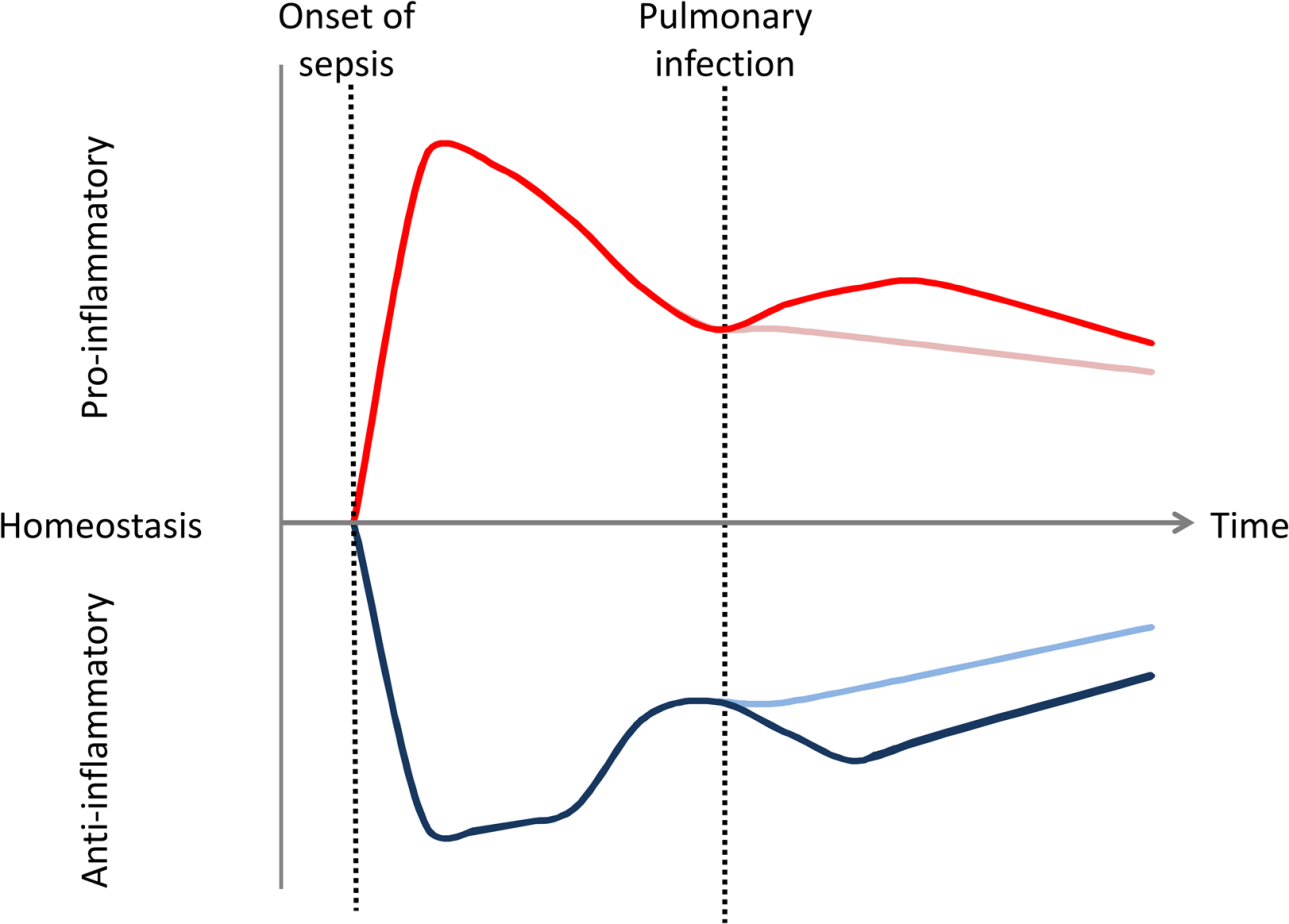
Theoretical potential inflammatory responses in mice double hit model. The observations made in this study show that pro and anti-inflammatory responses are concomitant in this model as in human. Despite immunoparalysis, intratracheal secondary infection due to *P*. *aeruginosa* reactivates slightly pro- and anti-inflammatory processes. Inflammatory response is represented by Red (pro) and blue (anti-inflammatory) lines. Dark lines represent initial inflammatory response and response after secondary infection whereas light lines represent the inflammatory response without secondary pulmonary infection.

At the moment of the intratracheal instillation of *P*. *aeruginosa* (D5), TNF and sTNFrs came back to initial values (Sham or CLP), whereas IL-6 and IL-10 remained higher in CLP as compared to Sham mice. Cytokine measurement after *Pseudomonas* lung administration shows reactivation of pro- and anti-inflammatory responses. Especially, TNF alpha was undetectable in most animals just before instillation (D5) and increased after the insult. This reactivation depends on bacterial load and animal status (CLP or Sham) as IL-6 and IL-10 concentrations rose concomitantly with administered CFU and were higher in CLP mice. However, at D7, CLP mice IL-6 levels seem to be lower at 10^8^CFU as compared to 2.10^7^ and 5.10^6^ CFU. This diminution could be explained because 40% of CLP mice, but no Sham, died before D7. As IL-6 is an associated risk factor for mortality [3], by the time of blood collection mice with the highest IL-6 levels were presumably dead. Regarding these results, both pro- and anti- inflammatory responses are reactivated and occur simultaneously after secondary pulmonary infection. Yet, this response is not as strongly triggered as after CLP (Fig 8).

Interestingly, this slight reactivation of the immune response, highlighted by the release of both pro- and anti-inflammatory mediators occurs despite immune defect at the time of intratracheal instillation. Five days after CLP we actually highlighted some alterations of T cells and monocytes’ response as described in the Review by R. Hotchkiss (2). We showed alterations in T cell proliferative response and monocyte TNFα production capacity in CLP mice’s spleen. The number of total T cells diminishes while the Treg subset of T cells increases. This decreasing T cells count can be put in parallel with lymphopenia which is correlated with the development of nosocomial infection in septic patients [28]. Moreover, T cells decreased proliferation is a component of immunoparalysis. Besides, Monneret *et al*. observed a strong correlation between increased Treg / effector ratio measured in whole blood after septic shock and decreased proliferative response of lymphocytes after mitogenic stimulation. Whereas total splenic T cells count is diminished on the fifth day post CLP, our results show that the Treg subset of T cells increases in accordance with Monneret et *al*. studies [29]. This observed expansion of splenic regulatory T cells may be another mechanism for immunosuppression as evidenced in human [10] since these lymphocytes are considered suppressive cells [10]. This expansion may be, in part, due to the elevation of IL-10, a cytokine responsible for the differentiation of CD4+ T cell into Treg rather than proliferation [30]. We observed impaired monocyte TNFα production capacity in CLP mice’s spleen after LPS stimulation. In parallel, after secondary *Pseudomonas* (gram negative bacteria) infection, mice exhibit similar reactivation of pro-inflammatory mediators such as TNFα in CLP and Sham. This discrepancy between ex vivo and in vivo results may highlight the importance of immune response compartmentalization in sepsis [22].

So, we demonstrated the homology between our first septic assault and the immune disorders occurring in septic patients. IL-10 and sTNFr secretion at the onset of sepsis, T-cells decreased count and proliferation and Treg cells up-regulation are consistent with what has been observed in humans. As described by Heidecke *et al*. working on T lymphocytes function to Weighardt *et al*. assessing cytokines production by LPS-stimulated monocytes, we came to the same conclusion that immunosuppression is primary rather than a compensatory response to sepsis [31,32].

In previously described mice double-hit models, the moment of secondary infection varies: Steinhauser *et al*. [18], Muenzer *et al*. [17] and Pène *et al*. [19] induce pneumonia 24h, 3 days or 8 days after CLP respectively. Delano *et al*. showed that the moment of secondary infection is also important and determines the percentage of survival [33]. In our study, the immune status of CLP mice was assessed at day 5, at the moment chosen for intratracheal secondary infection. Most nosocomial pneumonias occur between 3 and 5 days post hospitalization. The rational for choosing the fifth day is that mice mortality occurs until day 5. After 5 days all CLP mice survive suggesting that, in case of secondary infection, further mortality would not be due to initial CLP (Fig 5, PBS-instilled mice). In hospital, because of these acquired various immune defects, septic patients who survive the initial few days have a decreased capacity to overcome secondary infectious challenges and may have unresolved septic foci at time of death [34]. In our model, while all Sham mice survive, only 50% of CLP mice challenged with *Pseudomonas aeruginosa* (intratracheal route) at 2.10^7^ CFU are still alive after 8 days. Death occurs during the first 4 days after instillation. These results show that CLP mice are sensitized to secondary infection as it could be expected from their immune status. These results are consistent with previous clinical studies that showed a link between altered immune function and patient’s susceptibility to secondary infections [35,36]. However, the lethality of the secondary infection depends on the bacterial load. If the load is too high (10^8^ CFU), all mice die whether or not they have a previous CLP. Then, for high bacterial load, mortality is mostly due to *Pseudomonas* pneumonia itself rather than sepsis-induced immunodeficiency. If the load is too low, almost all the mice survive whether they are septic or not (only 10% mortality in the CLP group). These results point out the difficulty to standardize animal models in sepsis. More particularly, bacterial culture conditions and CFU measurement are very important in double-hit models. When mice are instilled with *Pseudomonas* the exact load is not well known. Culture growth may be determined using optical density (OD600), which is rather a measure of the light scattered by the bacterial suspension, which manifests itself as absorbance, than a number of viable bacteria. After mice intratracheal instillation the inoculum is serially diluted in order to determine bacterial cell count (CFU/ml) so that CFU is well known only the day after instillation. Moreover this measurement method is operator dependant and not very precise [37]. For these reasons, in a sepsis double-hit model, all the bacterial growth conditions must be carefully engineered before secondary infection to minimize the risk of error since a slight bacterial load variation can dramatically change the outcome of secondary infection. Culture conditions (amount, medium, temperature, duration of culture), determining the OD-CFU correlation when bacteria are instilled, using triplicate plates for CFU measurement are some of the conditions that must be optimized before performing secondary intratracheal instillation. These results also suggest that, in human, secondary infection could be dependent on bacterial load. Therefore, estimation of the bacterial load in septic patient, especially for nosocomial bacteria in ventilated patients, may be an indicator of the disease prognosis and reduction of microbial colonization represents a relevant therapeutic option (eg:. subglottic suction, reverse Trendelenberg’s (head up) position, reduced oropharyngeal colonization, …) [38].

The increased mortality due to intratracheal instillation seems to be associated with systemic bacterial dissemination. Actually, all mice survived in groups with no bacterial dissemination (Sham at 5.10^6^ and 2.10^7^ CFU or CLP at 5.10^6^ CFU) whereas mortality occurs in groups in which *Pseudomonas* was found in liver or spleen (Sham at 10^8^ CFU and CLP 2.10^7^ CFU and 2.10^8^ CFU). At 2.10^7^ CFU mortality and bacteria dissemination concerned only septic mice showing a higher susceptibility to nosocomial infection for CLP mice. After 7 days, in surviving mice, *Pseudomonas* was found in one CLP mice but no longer in Sham lungs suggesting a decreased pulmonary bacterial clearance as it has been already shown [39]. We couldn’t highlight any correlation between individual cytokine measurement and the intensity of bacterial dissemination after intratracheal infection.

This two-hit model mimics a classical scenario where patients in ICU with an intra-abdominal infection develop secondary *Pseudomonas aeruginosa* pneumonia, which is one of the most common causes of nosocomial pneumonia. We demonstrated that the combination of sub-lethal peritonitis followed by *Pseudomonas aeruginosa* pneumonia increases mortality. This mortality depends on the dose of instilled bacteria and is associated with systemic dissemination. After CLP, pro- and anti-inflammatory responses occur early and simultaneously. At the time of the secondary infection, despite observed immune defects in mice, pro- and anti- inflammatory responses are slightly reactivated depending on bacteria dose.

These results suggest that, in human, secondary infection might be dependent on bacterial load. Therefore, diminishing bacterial load or lung colonisation in septic patient may be a way to improve survival after secondary *Pseudomonas aeruginosa* pneumonia. Moreover, in previously septic ventilated patients, concomitant estimation of bacterial load (Brocho Alveolar Lavage, tracheal aspiration…) and systemic cytokine concentrations might be a prognosis factor to assess.

## Supporting Information

S1 Fig Figure explaining the conduction of our CLP procedure. 1) Laparotomy: A 1 cm midline cut is made into the skin only, approximately 0.5–1 cm away from xiphoid process. Then another 1 cm midline cut through the abdominal musculature into peritoneum is performed. 2) Isolation of cecum. Cecum is identified, isolated and gently exteriorized with a a non- crushing forceps. 3) Ligation of the cecum. Cecum is ligated on its external third (30% ≈ 0.8 to 1cm depending on the caecum size) with a non absorbable silk surgical suture (USP3/0, EP 2). 4) Puncture of the cecum. Cecum is punctured twice with a 21-gauge needle to create two single holes (this is not a through and through puncture). After removing the needle, a small amount (droplet) of feces from penetration holes was extruded to ensure patency. The wound is sutured in layers. First, muscles are sutured using an absorbable polyglycolic acid suture (USP3/0, EP 2). Then, skin is closed with Michel wound clips (7.5 mm).
(PDF)

S2 Fig Figure illustrating lungs histology after secondary infection. Methods: Histologic analysis of hematoxylin and eosin (H&E)-stained lung specimens was performed to confirm the presence of pneumonia 48h and 7 days after intra-tracheal administration of P. aeruginosa (2.107 CFU). Briefly, lungs were removed and fixed by intra- tracheal infusion of paraformaldehyde (4%). They were kept in 4% paraformaldehyde at least 36h, dehydrated in successive bath with respectively 30, 50 and 70% of ethanol, embedded in paraffin, cut into 8μm-sections and stained with H&E. Results: Two days after Pseudomonas administration both CLP and Sham mice exhibited pneumonia as we observed intra-alveolar hemorrhage and massive inflammation, as extensive polymorphonuclear and mononuclear cell infiltration in both groups. Seven days after intra-trachal administration, alveolar infiltration diminished more in Sham operated mice than in CLP mice.
(PDF)

## Abbreviations

P. aeruginosa: Pseudomonas aeruginosa
I.P.: intraperitoneal
TNFα: tumor necrosis factor alpha
IL-6: interleukin 6
IL-10: interleukin 10
sTNFr: solutme tumor necrosis factor receptor
CLP: cecal ligation and puncture
PAMPs: pathogen associated molecular patterns
DAMPs: danger associated molecular patterns
T cell: T lymphocyte
Treg: regulatory T lymphocyte
ELISA: enzyme linked immune assay
VAP: ventilator-associated pneumonia
