# Supplementary material for: Mice Survival and Plasmatic Cytokine Secretion in a “Two Hit” Model of Sepsis Depend on Intratracheal Pseudomonas Aeruginosa Bacterial Load
Source: PLoS One. 2016 Aug 30;11(8):e0162109. doi: 10.1371/journal.pone.0162109 (PMC5004855; doi:10.1371/journal.pone.0162109)
Supplement: S1 Fig — 1) Laparotomy: A 1 cm midline cut is made into the skin only, approximately 0.5–1 cm away from xiphoid process. Then another 1 cm midline cut through the abdominal musculature into peritoneum is performed. 2) Isolation of cecum. Cecum is identified, isolated and gently exteriorized with a a non- crushing forceps. 3) Ligation of the cecum. Cecum is ligated on its external third (30% ≈ 0.8 to 1cm depending on the caecum size) with a non absorbable silk surgical suture (USP3/0, EP 2). 4) Puncture of the cecum. Cecum is punctured twice with a 21-gauge needle to create two single holes (this is not a through and through puncture). After removing the needle, a small amount (droplet) of feces from penetration holes was extruded to ensure patency. The wound is sutured in layers. First, muscles are sutured using an absorbable polyglycolic acid suture (USP3/0, EP 2). Then, skin is closed with Michel wound clips (7.5 mm). (PDF) [file pone.0162109.s001.pdf]

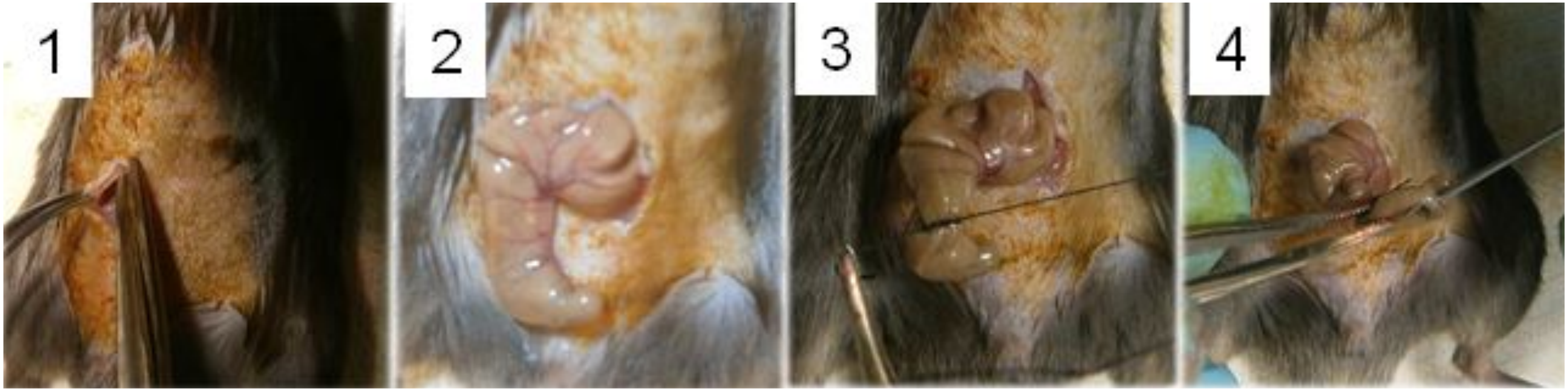

S1 Fig: Conduction of CLP

1) Laparotomy: A 1 cm midline cut is made into the skin only, approximately 0.5-1 cm away from xiphoid process. Then another 1 cm midline cut through the abdominal musculature into peritoneum is performed.

2) Isolation of cecum. Cecum is identified, isolated and gently exteriorized with a a non-crushing forceps.

3) Ligation of the cecum. Cecum is ligated on its external third (30% ≈ 0.8 to 1cm depending on the caecum size) with a non absorbable silk surgical suture (USP3/0, EP 2).

4) Puncture of the cecum. Cecum is punctured twice with a 21-gauge needle to create two single holes (this is not a through and through puncture). After removing the needle, a small amount (droplet) of feces from penetration holes was extruded to ensure patency.

The wound is sutured in layers. First, muscles are sutured using an absorbable polyglycolic acid suture (USP3/0, EP 2). Then, skin is closed with Michel wound clips (7.5 mm).
