# Supplementary material for: Mice Survival and Plasmatic Cytokine Secretion in a “Two Hit” Model of Sepsis Depend on Intratracheal Pseudomonas Aeruginosa Bacterial Load
Source: PLoS One. 2016 Aug 30;11(8):e0162109. doi: 10.1371/journal.pone.0162109 (PMC5004855; doi:10.1371/journal.pone.0162109)
Supplement: S2 Fig — Methods: Histologic analysis of hematoxylin and eosin (H&E)-stained lung specimens was performed to confirm the presence of pneumonia 48h and 7 days after intra-tracheal administration of P. aeruginosa (2.107 CFU). Briefly, lungs were removed and fixed by intra- tracheal infusion of paraformaldehyde (4%). They were kept in 4% paraformaldehyde at least 36h, dehydrated in successive bath with respectively 30, 50 and 70% of ethanol, embedded in paraffin, cut into 8μm-sections and stained with H&E. Results: Two days after Pseudomonas administration both CLP and Sham mice exhibited pneumonia as we observed intra-alveolar hemorrhage and massive inflammation, as extensive polymorphonuclear and mononuclear cell infiltration in both groups. Seven days after intra-trachal administration, alveolar infiltration diminished more in Sham operated mice than in CLP mice. (PDF) [file pone.0162109.s002.pdf]

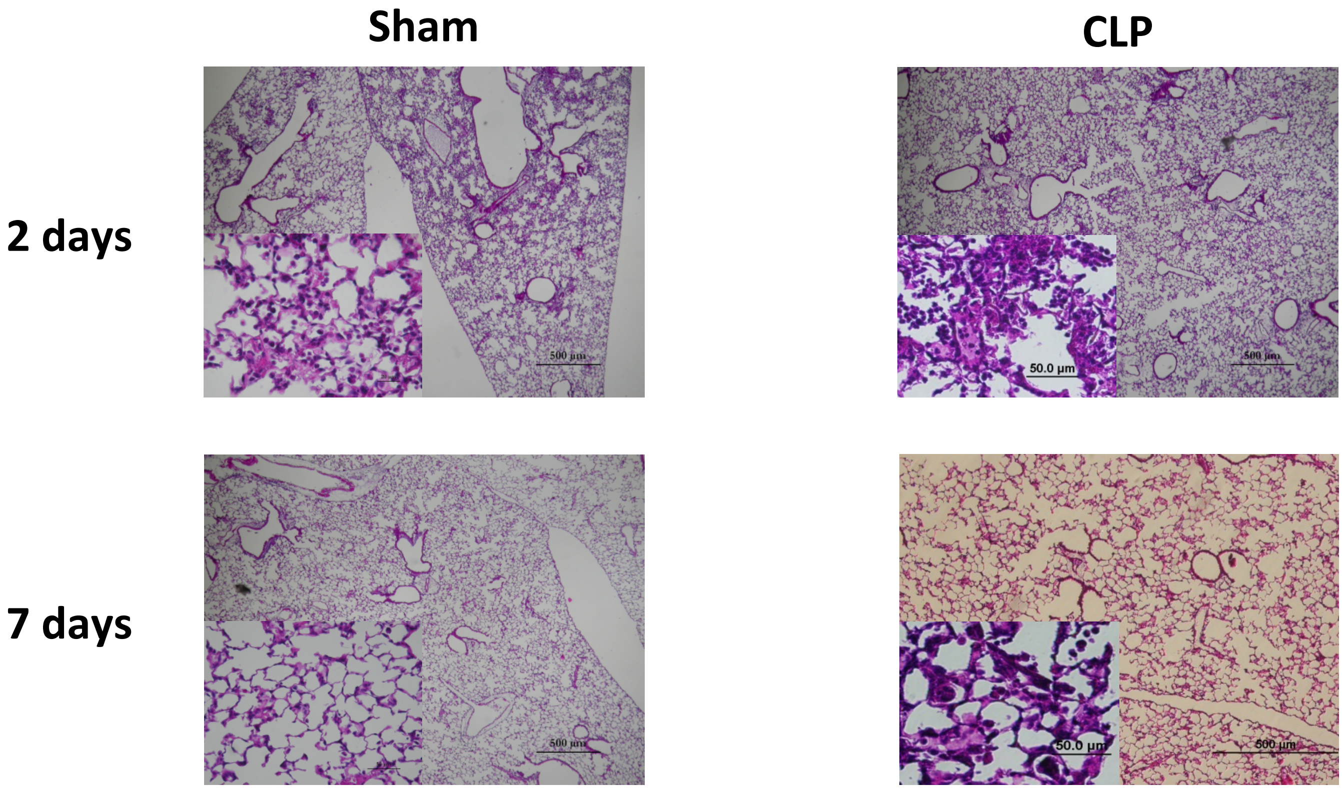

S2 Fig: Lungs histology after secondary infection

Methods: Histologic analysis of hematoxylin and eosin (H&E)-stained lung specimens was performed to confirm the presence of pneumonia 48h and 7 days after intra-tracheal administration of *P. aeruginosa* (2.107 CFU). Briefly, lungs were removed and fixed by intra-tracheal infusion of paraformaldehyde (4%). They were kept in 4% paraformaldehyde at least 36h, dehydrated in successive bath with respectively 30, 50 and 70% of ethanol, embedded in paraffin, cut into 8µm-sections and stained with H&E.

Results: Two days after Pseudomonas administration both CLP and Sham mice exhibited pneumonia as we observed intra-alveolar hemorrhage and massive inflammation, as extensive polymorphonuclear and mononuclear cell infiltration in both groups. Seven days after intra-trachal administration, alveolar infiltration diminished more in Sham operated mice than in CLP mice.
